# The characteristics of the intestinal bacterial community from *Oreochromis mossambicus* and its interaction with microbiota from artificial fishery habitats

**DOI:** 10.1186/s12862-023-02120-2

**Published:** 2023-05-08

**Authors:** Sheng Bi, Han Lai, Dingli Guo, Huadong Yi, Haiyang Li, Xuange Liu, Qiuxian Chen, Jiahui Chen, Zhilun Zhang, Xuchong Wei, Guifeng Li, Guorong Xin

**Affiliations:** 1 School of Agriculture, Shenzhen Campus of Sun Yat-sen University, Guangdong, 518107 China; 2 State Key Laboratory of Biocontrol, Southern Marine Science and Engineering Guangdong Laboratory (Zhuhai), Guangdong Provincial Key Laboratory for Aquatic Economic Animals, School of Life Sciences, Sun Yat-Sen University, Guangzhou, 510006 China; 3 Guangdong Provincial Engineering Technology Research Center for Healthy Breeding of Important Economic Fish, Guangzhou, 510006 China

**Keywords:** Hydrobiology, 16S rRNA, tilapia, Microbial ecology

## Abstract

**Background:**

Artificial habitats can allow many fish to flock together and interact and have been widely used to restore and protect fishery resources. The piece of research intends to elucidate the relationship of microbial communities between tilapia (*Oreochromis mossambicus*) intestines and artificial fishery habitats (water and sediments). Hence, 16 S rDNA sequencing technology was used to study the bacterial communities from intestines, water, and sediments.

**Results:**

The results showed that the tilapia intestines had the lowest richness of Operational Taxonomic Units (OTUs) and the lowest diversity of the bacterial community compared to water and sediments. The intestine, water, and sediment microbial communities shared many OTUs. Overall, 663 shared OTUs were identified from the tilapia intestines (76.20%), the surrounding water (71.14%), and sediment (56.86%) in artificial habitats. However, there were unique OTUs that were detected in different sample types. There were 81, 77 and 112 unique OTUs observed in tilapia intestines, the surrounding water and sediment, respectively. Proteobacteria, Cyanobacteria, Actinobacteria, Firmicutes, Fusobacteria, and Bacteroidetes were the most common and dominant bacterial phyla between the tilapia intestines and habitats. In the two groups, the microbial communities were similar in the taxonomic composition but different in the abundance of bacterial phyla. Interestingly, Firmicutes increased, while Fusobacteria decreased in artificial habitats. These findings indicated that the artificial habitats had fewer effects on the water environment and indicated that the mode of artificial habitats could have an effect on the enriched bacteria in the tilapia intestines.

**Conclusions:**

This study analysed the bacterial communities of artificial habitats from the intestines, water, and sediments, which can explain the relationship between the tilapia intestines and habitats and strengthen the value of ecological services provided by artificial habitats.

## 1. Background

The *Oreochromis mossambicus* of tilapia has a high survival rate, strong disease resistance, adaptability to the environment, and rich protein content. Currently, it has become one of the main sources of animal protein [1–3]. Because of the extensive adaptability and strong fecundity in tilapias, *O. mossambicus* has become a dominant species in the Youjiang artificial habitats [4, 5]. Much research has been conducted on tilapia and fish-associated microbiota [6–8]. These studies have shown that fish-associated microbiota plays a crucial role in digestion, growth, and disease resistance [9–12]. For example, fish intestines are occupied by a variety of commensal and pathogenic microorganisms, which participate in the whole process of fish growth and development [13, 14]. Since fish live in the water environment, the composition and function of their intestinal microbiota will be strongly affected by their habitats [15]. In fact, fish intestine microbiota are enriched through those from the environment, thus completing the transfer process of microbiota from water to fish intestines [7, 16, 17]. It has been shown that the gut microbial compositions of fish, crabs, and shrimp are significantly affected by the surrounding habitats [18–20]. Most of these studies have been conducted by changing water conditions, feeding patterns, and increasing stress factors (e.g., varying temperature and dissolved oxygen). Indeed, it is rare to study the intestinal microbiota of tilapia based on complex artificial habitats. For tilapia living in artificial fishery habitats, the effects of habitat on host-related microbiota remain relatively unclear.

The role of an artificial fishery habitat is to imitate the characteristics of the natural habitat in water areas and to increase habitat heterogeneity under the condition of a single habitat [21]. Most research has clarified the role of artificial habitats in protecting fisheries, including enticing fish and improving fish abundance and biomass [22–26], providing spawning attachment substrates [27, 28], and offering a haven for juvenile fishes [29]. Additionally, when artificial habitats play these roles, they greatly affect the microbial communities in the fish intestines, water, and sediment. In recent years, researchers have conducted a few studies about the impact of artificial habitats on microbial diversity [30, 31]. However, studies on the relationship between the microbial communities of tilapia intestines and artificial fishery habitats are still rare. Although changes in feed nutrition are known to affect the environment and fish gut microbial composition [32–34], the differentiation of microbial communities between different host species remains to be clarified in unfed aquaculture systems.

In this research, we aimed to characterize the bacterial communities’ relationship in the tilapia intestines and artificial habitats. The results indicated the following: (1) the compositions of bacteria in the tilapia intestines, water, and sediment were similar, while the relative abundance of bacteria varied, and (2) the microbial composition of tilapia intestines changed significantly under the influence of artificial habitats, despite no changes detected in the microbial composition of the surrounding water. This study provides new pieces of evidence for the role of artificial fishery habitats and puts forward insights into the composition, diversity, and function of tilapia intestinal microbiota, which can be affected by surrounding habitats.

## 2. Results

### 2.1 Overview of the OTUs and diversity analysis

A total of 4,601,275 sequence reads were generated by 16 S rDNA sequencing of the samples. The sequences were clustered into 830, 615, 913, 969, 1130, and 1239 OTUs from the AI, CI, AW, CW, AS, and CS, respectively (Table 1). The total intestinal OTUs were significantly different between AI and CI. The sediment groups (AS; CS) had a similar result, but there was no significant difference between the water groups (AW; CW).

The alpha-diversity indices were determined at the OTUs level to evaluate the diversity of the bacterial communities in all groups (Table 1). The Shannon index was in the range of 4.34 to 5.52 in the intestines, 7.15 to 7.22 in the water, and 6.61 to 6.93 in the sediment. The Simpson index was used to estimate the bacterial community dominance within a range of 0.1573 to 0.0366 in intestines, 0.0147 to 0.0186 in water, and 0.0125 to 0.0428 in sediment. The coverage was always kept at 0.99. These results indicated that tilapia intestines had the lowest OTU abundance and bacterial community diversity compared with the sampled habitats. The Chao-1 index was used to estimate bacterial community richness which ranged from 794 to 855 phylotypes in intestines, 932 to 991 phylotypes in water, and 1266 to 1359 phylotypes in sediment. There were no significant differences in the Chao-1 index.

**Table 1:** Overview of the high-throughput read analysis, including total OTUs and diversity statistics

Groups	Intestines		Water		Sediment	
	AI	CI	AW	CW	AS	CS
Total OTUs (97%)	830 ± 33 a	615 ± 21 b	913 ± 56 c	969 ± 49 c	1130 ± 101 c	1239 ± 99 d
Diversity indices						
Shannon	5.52 ± 0.27 a	4.34 ± 0.14 b	7.15 ± 0.48 c	7.22 ± 0.26 c	6.61 ± 0.08 c	6.93 ± 0.14 c
Simpson	0.0366 ± 0.0021 a	0.1573 ± 0.0018 b	0.0186 ± 0.0003 c	0.0147 ± 0.0005 c	0.0428 ± 0.0058 a	0.0125 ± 0.0043 d
Chao-1	855 ± 124 a	794 ± 96 a	932 ± 36 b	991 ± 59 b	1266 ± 106 c	1359 ± 218 c
Coverage	0.99 ± 0.01	0.99 ± 0.01	0.99 ± 0.01	0.99 ± 0.01	0.99 ± 0.01	0.99 ± 0.01

The OTUs and the bacterial community diversity analysis of all samples from intestines (I), water (W), and sediment (S) in artificial habitats (AI, AW, and AS) and control areas (CI, CW, and CS) are shown. The means ± SD data of Table 1 in the same row with different letters differ significantly (P < 0.05)

**Fig. 1:**
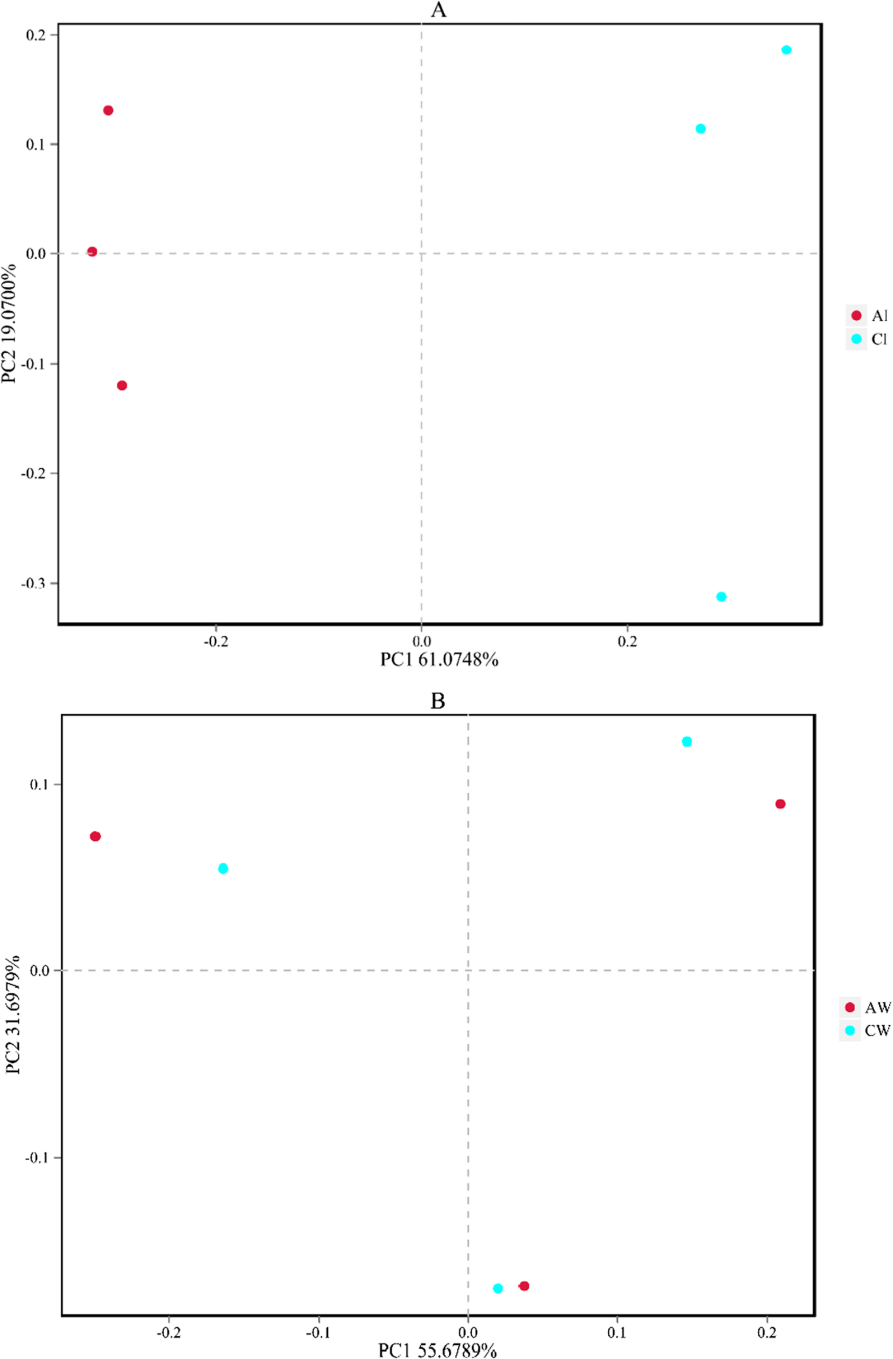

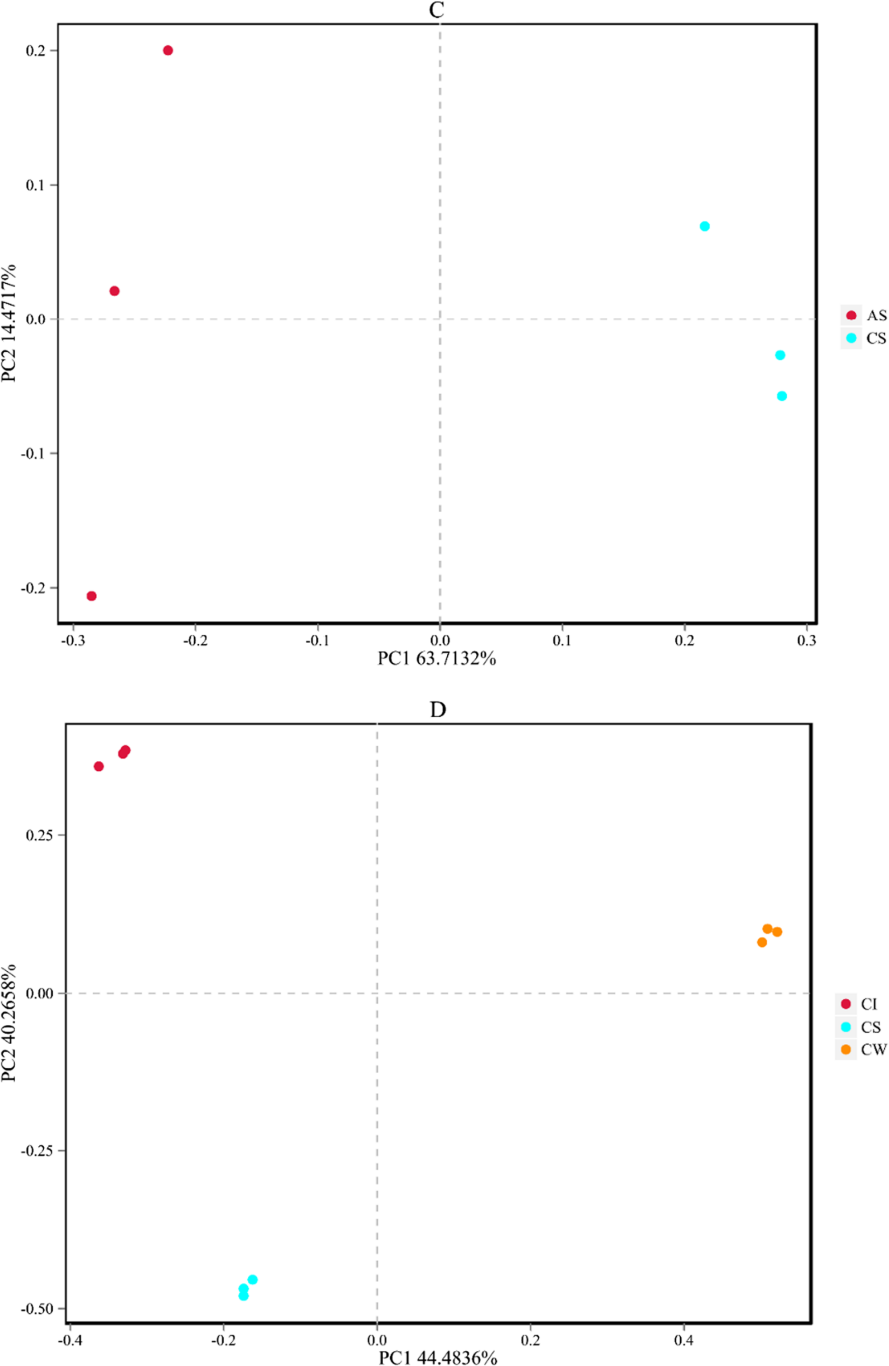

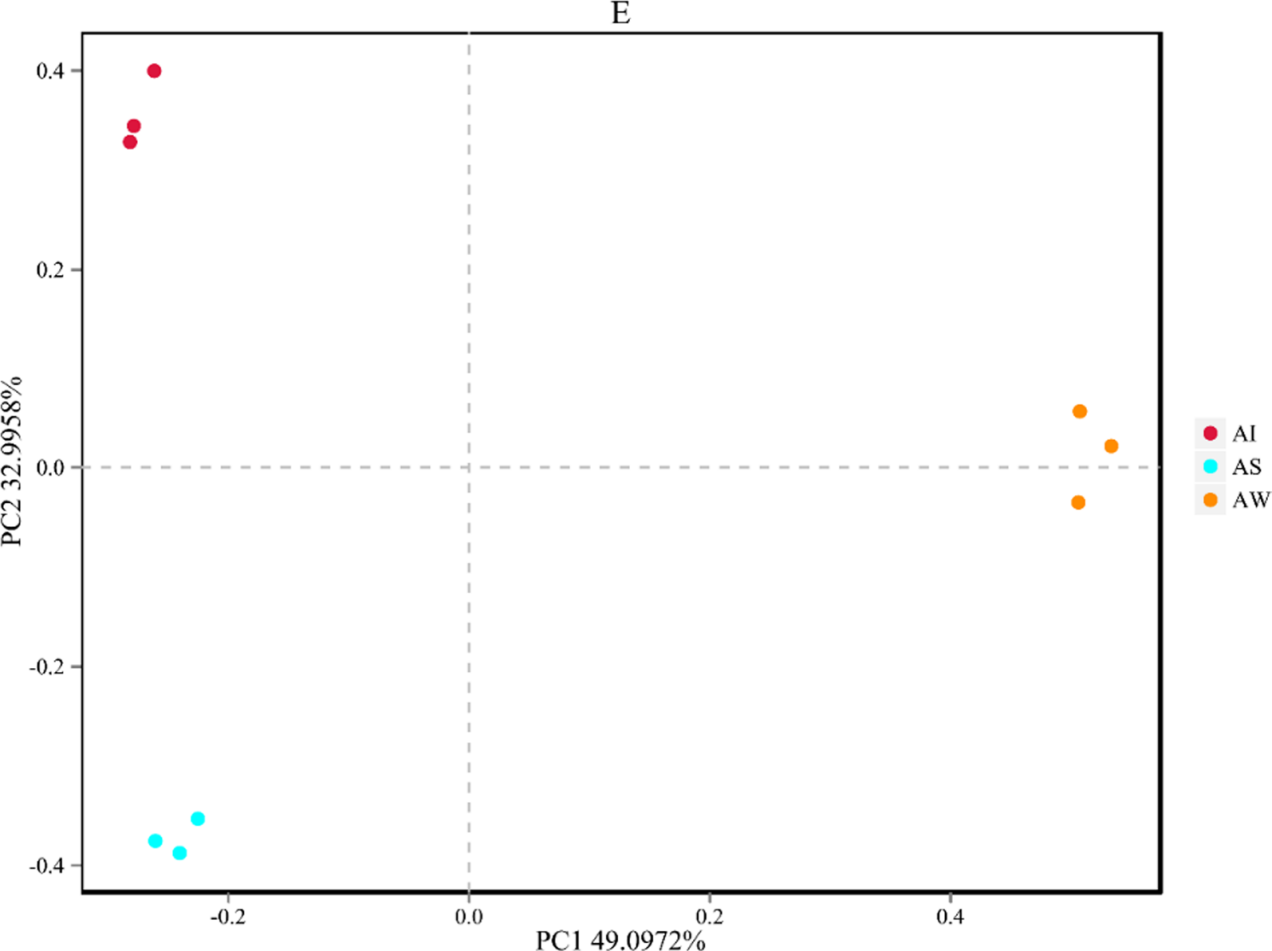
The PCoA in intestines, water, and sediment samples between artificial habitats and control areas. Figure **A**–**C** provides comparisons of intestines, water, and sediment within different habitats. Figure **D** shows the comparison of bacterial communities in control areas. Figure **E** shows the comparison of bacterial communities in artificial habitats. AI, intestines of tilapia in the artificial habitats; AS, sediment in the artificial habitats; AW, water in the artificial habitats; CI, intestines of tilapia in the control areas; CS, sediment in the control areas; CW, water in the control areas

Principal coordinate analysis (PCoA) by Bray-Curtis distances showed that bacterial communities from intestines and sediment were obviously divided (Fig. 1A and C), while bacterial communities in water were similar between the two habitats, with a certain degree of convergence (Fig. 1B). Moreover, the analysis revealed a stable clustering of microbial communities in habitats (Fig. 1D, E). These results revealed that the differences between fish gut groups were high and that the similarities between habitat groups were stable.

### 2.2 Taxonomic composition

**Fig. 2:**
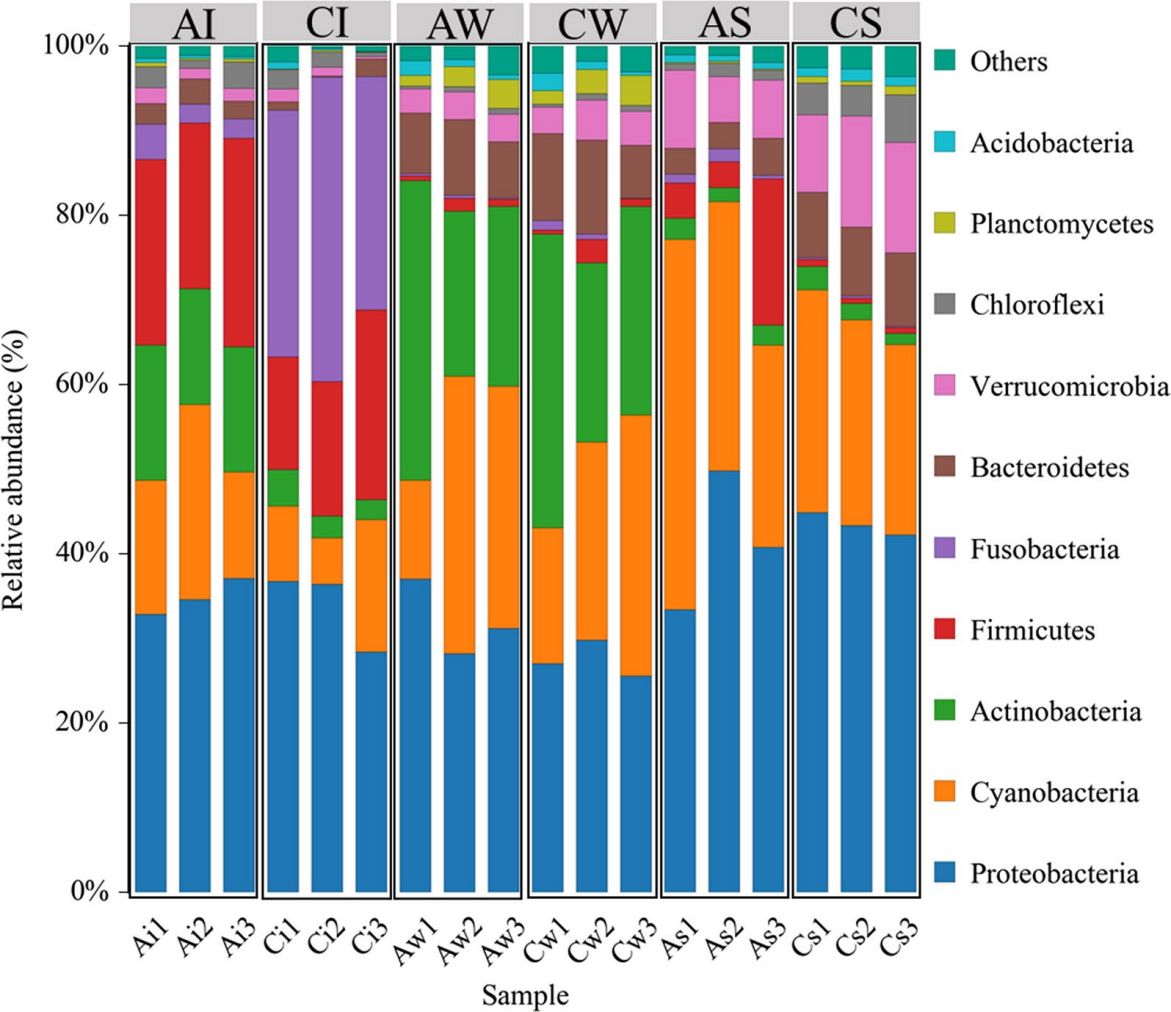
Bacterial community composition at the phylum level in tilapia intestines, water, and sediment

The dominant bacterial phyla in the groups were Proteobacteria, Cyanobacteria, Actinobacteria, Firmicutes, etc. (Fig. 2). It was similar in taxonomic composition to the microbial communities, but the bacterial phyla distribution was different in abundance. In artificial habitats, the most abundant phylum was Proteobacteria in tilapia intestines, water, and sediment, with a relative abundance of 34.85% in the AI groups, 32.19% in the AW groups, and 41.36% in the AS groups. The second most abundant phylum was Cyanobacteria, with a relative abundance of 17.17% in the AI groups, 24.31% in the AW groups, and 33.15% in the AS groups. In control areas, Proteobacteria was also the most abundant phylum in tilapia intestines and habitats, with a relative abundance of 33.87% in the CI groups, 27.51% in the CW groups, and 43.52% in the CS groups. The second most abundant phylum was also Cyanobacteria, with a relative abundance of 10.12% in the CI groups, 23.42% in the CW groups, and 24.37% in the CS groups. In the tilapia intestine, Proteobacteria, Cyanobacteria, Actinobacteria, Firmicutes, and Fusobacteria were the five most dominant phyla in both habitats. The Fusobacteria abundance in tilapia intestines was higher when compared to that found in the surrounding habitats.

In artificial habitats, the closest relatives were separated into 218 genera of Proteobacteria, 55 genera of Cyanobacteria, 60 genera of Actinobacteria, 65 genera of Firmicutes, 63 genera of Bacteroidetes, and 124 genera of other phyla (Table 2). Meanwhile, in control areas, the closest relatives were divided into 215 genera of Proteobacteria, 53 genera of Cyanobacteria, 2 genera of Fusobacteria, 55 genera of Actinobacteria, 61 genera of Firmicutes, and 199 genera of other phyla (Table 3).

**Table 2:** Genus level differences in dominant phyla in artificial habitats

Phylum	Numbers of genera	Genera
Proteobacteria	218 genera	*Rhodopila, Roseococcus, Rubritepida, Brevundimonas, Phenylobacterium*, etc.
Cyanobacteria	55 genera	*Limnothrix, Chlorotetraedron_incus, Leptolyngbya_PCC-6306*, etc.
Actinobacteria	60 genera	*Ilumatobacter, Brevibacterium, hgcI_clade, Brachybacterium*, etc.
Firmicutes	65 genera	*Bacillus, Fictibacillus, Allobaculum*, etc.
Bacteroidetes	63 genera	*Bacteroides, Alloprevotella, Runella*, etc.
Others	124 genera	*Bryobacter, Brevifollis, Luteolibacter*, etc.

**Table 3:** Genus level differences in dominant phyla in control areas

Phylum	Numbers of genera	Genera
Proteobacteria	215 genera	*Rhodopila, Roseococcus, Rubritepida, Methylobacterium, Arenimonas*, etc.
Cyanobacteria	53 genera	*Limnothrix, Prochlorococcus_MIT9313, Leptolyngbya_PCC-6306*, etc.
Fusobacteria	2 genera	*Cetobacterium, Hypnocyclicus*
Actinobacteria	55 genera	*Ilumatobacter, Micrococcus, Microbacterium, hgcI_clade*, etc.
Firmicutes	61 genera	*Blautia, Fictibacillus, Lactobacillus*, etc.
Others	199 genera	*Sulfurospirillum, Helicobacter, Gemmatimonas*, etc.

**Fig. 3:**
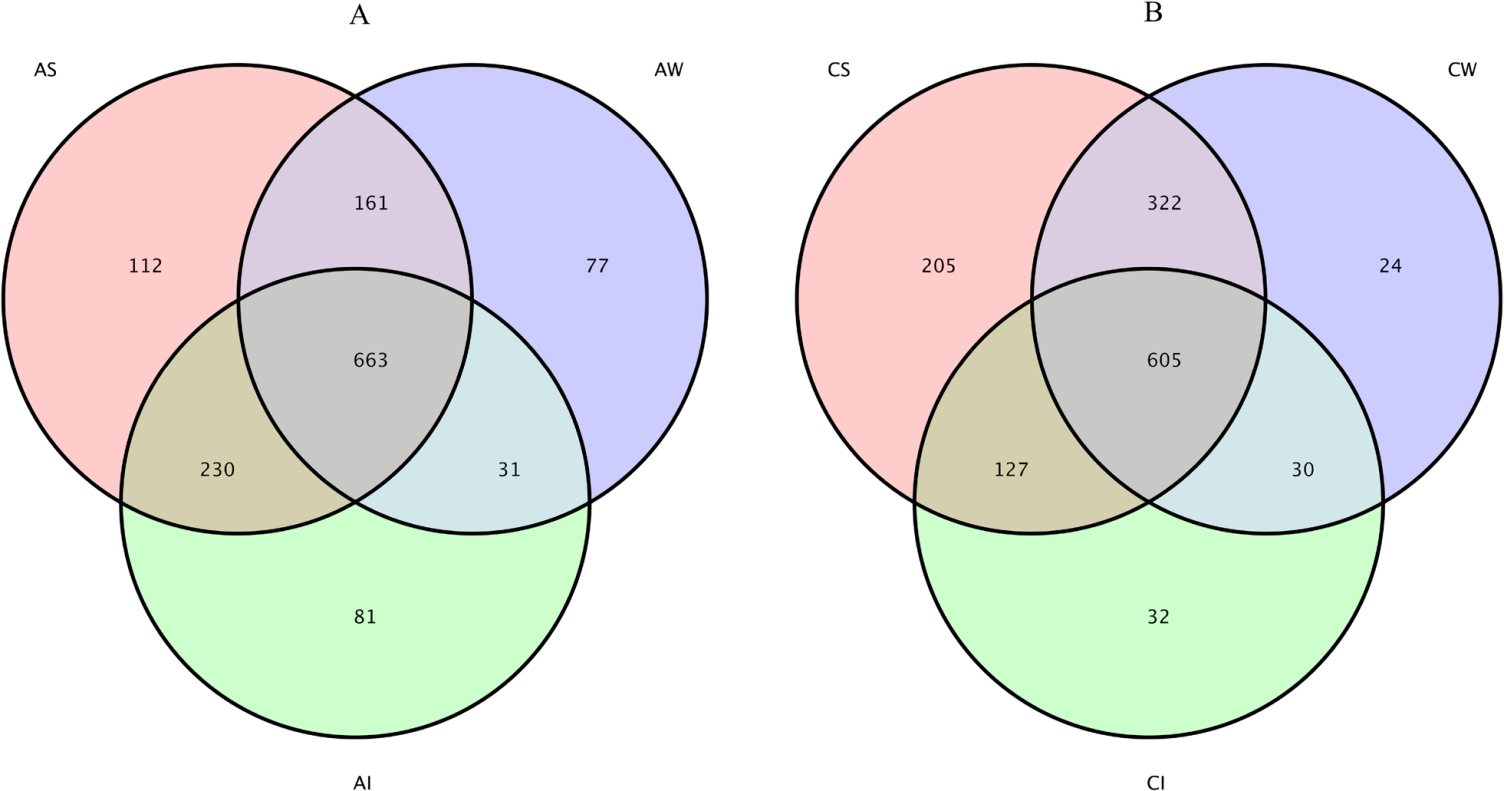
**A** Venn plot showing OTUs overlap of the AI, AS and AW groups; **B** Venn plot showing overlap of the CI, CS, and CW groups. AI, intestines in artificial habitats; AS, sediment in artificial habitats; AW, water in artificial habitats; CI, intestines in control areas; CS, sediment in control areas; CW, water in control areas

The bacterial OTUs in the tilapia intestines and habitats were investigated between two modes of habitats and are shown quantitatively in Fig. 3. In the mode of artificial habitats, a total of 663 collective OTUs were found among tilapia intestines, the surrounding water, and sediment (Fig. 3A), with an average of 76.20%, 71.14%, and 56.86% shared OTUs for the AI, AW and AS groups, respectively. In addition, 81, 77, and 112 unique OTUs were found in the intestines, water, and sediment, respectively.

In the control areas, 605 collective OTUs were detected in the intestines, water, and sediment (Fig. 3B). The number of shared OTUs accounted on average for 65.97%, 61.67%, and 48.05% of the total bacterial communities in the CI, CW, and CS groups, respectively. In addition, 81, 77 and 112 unique OTUs were observed in the intestines, surrounding water, and sediment, respectively.

### 2.3 Effects of artificial habitats on the bacterial communities of tilapia intestines, water, and sediment

This piece of research explored the effect of artificial habitats on the bacterial community, and the linear discriminant analysis effect size (LEfSe) method was used to identify differently enriched taxa between the artificial habitats (AH) and control areas without artificial structures (CW). At the taxonomic level, LEfSe could effectively analyse the data [35]. When the linear discriminant analysis (LDA) value setting was 2, there were 658 bacterial groups. To make the cladograms clearer, the LDA value was set to 4 [30, 36].

**Fig. 4:**
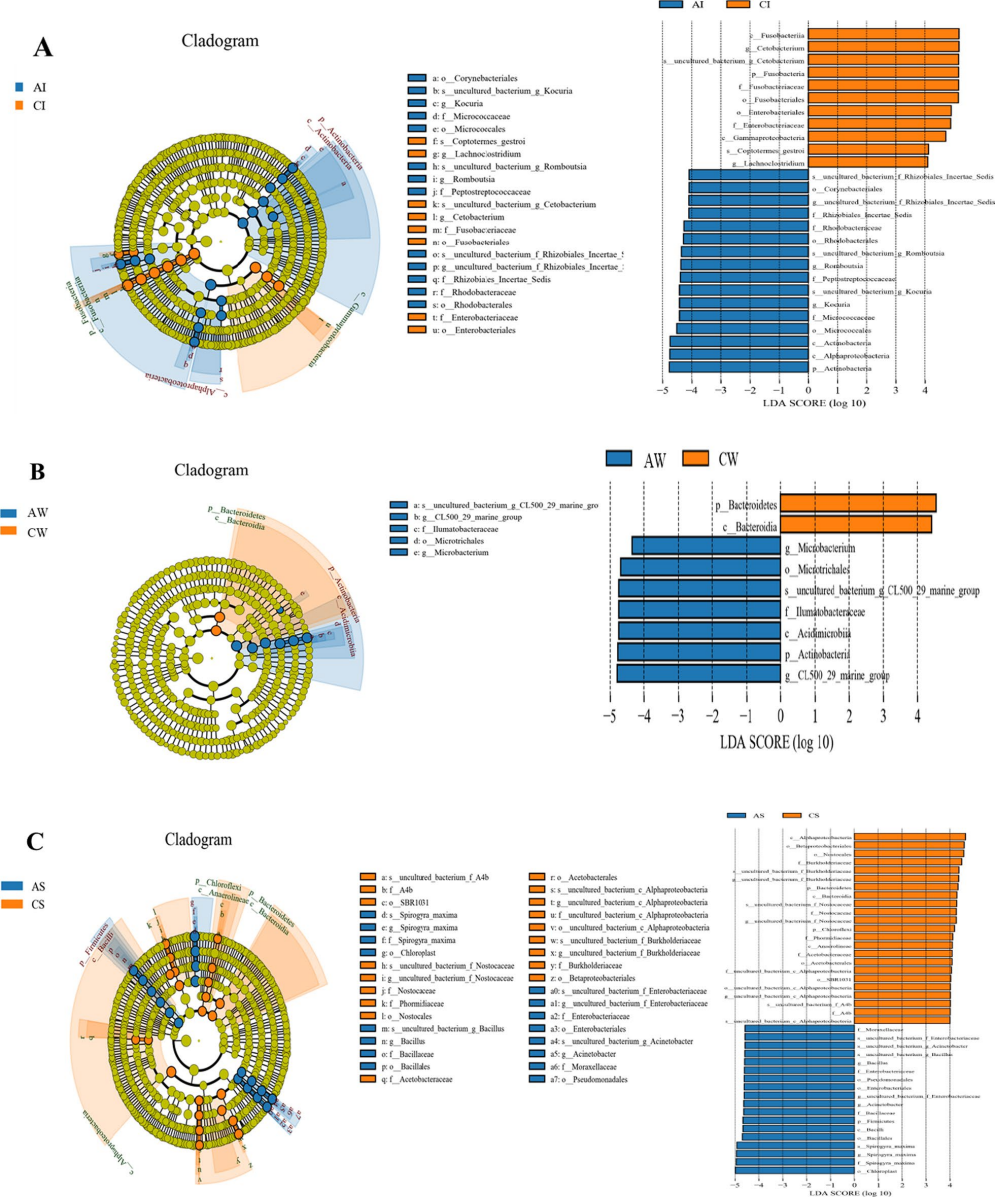
LEfSe analysis (*P* < 0.05) of intestinal microflora (**A**), water (**B**), and sediment (**C**) between artificial and control habitats

The dominant species in the bacterial community changed significantly (Fig. 4), while the LEfSe analysis revealed that artificial habitats influenced some biomarkers (*P* < 0.05, LDA > 4.0). There were 16 biomarkers enriched in intestines from the AI (Fig. 4A), including Actinobacteria, Alphaproteobacteria, Micrococcales, Peptostreptococcaceae, and *Rhodobacteraceae*. Moreover, there were 11 biomarkers enriched in intestines from the CI group (Fig. 4A), including Fusobacteria, Fusobacteriia, Fusobacteriales, Fusobacteriaceae, and *Cetobacterium*. The water samples had the lowest number of biomarkers. There were 7 bacteria biomarkers enriched in water samples from the AW groups (Fig. 4B), including Actinobacteria, Microtrichales, Ilumatobacteraceae, *Microbacterium*, and *CL500_29_marine_group* (at the genus level), among others. However, there were only two bacteria biomarkers enriched in water samples from the CW group (Fig. 4B), namely, Bacteroidetes and Bacteroidia. The sediment samples had the highest number of biomarkers. There were 18 bacteria biomarkers enriched in sediment samples from the AS group (Fig. 4C), including Firmicutes, Bacilli, Bacillales, Pseudomonadales, Bacillaceae, and others. Moreover, there were 23 bacteria biomarkers enriched in sediment samples from the CS (Fig. 4C), including Alphaproteobacteria, Bacteroidetes, Betaproteobacteriales, Nostocales, Burkholderiaceae, and others.

## 4. Discussion

It is generally believed that the microbiota plays a crucial role in host nutrition and health [37–39]. Increasing evidence indicates that environmental microbes are associated with microbial digestive system diseases in the intestines of aquatic organisms (such as fishes, crabs, and shrimp) [17, 40, 41]. Studies to investigate the bacterial community composition in sediment [6], water [17], and tilapia intestines [7] have been previously conducted. Nevertheless, studies on the relationship of microbial communities between the tilapia intestines and artificial fishery habitats are still rare. Here, we attempt to elucidate the relationship between bacterial communities in intestines and habitats and then propose a method for studying bacterial communities in unfed aquaculture ecological systems. Moreover, we compared the bacterial community between fish intestines and the surrounding environment in artificial habitats.

The Shannon index was used to estimate the bacterial diversity, and the Simpson index was used to confirm it. There was a lower diversity in tilapia intestines when compared to the surrounding habitats (*P* < 0.05) (Table 1). It has been shown that the intestinal bacterial diversity is lower than that in water and sediment [6]. Similar results have been found for other aquatic organisms, including shrimps (*Litopenaeus vannamei* and *Litopenaeus stylirostris*) and crabs (*Eriocheir sinensis*) [19, 20, 42]. This study hypothesized that habitats would have a higher diversity compared to the intestines of tilapia due to environmental differences between habitats. However, the results showed that not all microbes in habitats could be ingested by the intestines of tilapia (Fig. 3). In fact, the tilapia intestines were less aerobic than water and sediment and had immunological factors that may select specific types of bacteria [43–46]. The intestines, water, and sediment exhibited marked similarities in the field of shared OTUs, core taxa, and composition. Shared OTUs were found for tilapia intestines, the surrounding water, and sediment, indicating that there are considerable microbes coexisting among these samples (Figs. 2 and 3). In the artificial habitat, the dominant bacterial phyla in the groups were Proteobacteria, Cyanobacteria, Actinobacteria, Firmicutes, and so on. This was similar to the composition of bacteria in the tilapia intestines, water, and sediment, while the relative abundance of bacteria varied. Taking Firmicutes and Fusobacteria as examples, the relative abundance of bacteria in the intestines was significantly higher than that in the habitats. Firmicutes increased while Fusobacteria decreased in artificial habitats when compared to those of the control areas. Previous research has shown that Firmicutes can promote caloric extraction of ingested food substances and energy regulation [47, 48], while Fusobacteria is a potential intestinal pathogen that can cause inflammation and abdominal infection [49, 50]. Changes in tilapia intestinal microflora showed that artificial habitats could play a better role in promoting the growth of tilapia and decreasing the risk of infection.

The main difference between samples from different environments (intestine, water, and sediment) regardless of habitat was the relative abundance. Furthermore, there was a discrepancy in enriched bacteria within the same sample type under different habitats. LEfSe (LDA > 4.0) analysis found that the bacterial communities of the tilapia intestine had significant changes (Fig. 4). The relative richness of Actinobacteria (at the phylum level) was higher in AI than in CI, while Fusobacteria (at the phylum level) was significantly higher in CI than in AI (Fig. 4A). However, the microbial community changes in habitat samples were strikingly different under the two habitat modes. The water samples had the lowest numbers of biomarkers (Fig. 4B), while sediment samples had the highest (Fig. 4C). These results showed that the artificial habitat had a lower impact on the water environment and indicated that artificial habitats could affect enriched bacteria in the tilapia intestines. It is generally believed that the intestinal microflora of freshwater fishes comes from the environment [51], and differences in intestinal microflora among different habitats may be caused by bacteria gathered in specific habitats [36, 52].

Findings from this piece of research were consistent with results previously obtained by other researchers that showed a similar bacterial community composition between fish intestines and surroundings [6, 53]. In addition, the interaction between the intestinal bacterial community and the surrounding environment was associated with aquatic animal diseases [17, 54, 55]. Some potential pathogenic bacteria were also identified in the tilapia intestines and surroundings, such as *Flavobacterium* and *Pseudomonas* in water and *Vibrio* in the intestines, which may be linked to aquatic animal diseases. Indeed, *Vibrio* was shown to amplify the chance of Hepato pancreas necrosis syndrome (HPNS) outbreaks [40]. Our results showed that taxa and microbial diversities were significantly different between the tilapia intestines and habitat, demonstrating that the habitat mode may also affect the composition of the bacterial community in fish intestines and the surrounding environment. Altogether, the relationship between fish intestines and the surrounding environment still needs further investigation in addition to the influence of habitat change, including the role of environmental factors and food intake.

## 5. Conclusions

This research represented an attempt to study bacterial communities related to tilapia intestines, water, and sediment in artificial fishery habitats. In short, we generated profiles of microbial communities in tilapia intestines, water, and sediment. It was evident that the microbial community composition was similar, but the bacterial distribution abundance was different between habitats. The microbial composition of tilapia intestines changed significantly under the influence of artificial habitats; however, the microbial composition of water was unaffected. Overall, we provide insights into the relationship between bacterial communities in intestines and habitats. This study provides new scientific evidence for the role of artificial fishery habitats and provides insights into the composition, diversity, and function of tilapia microbiota, which strengthens the value of ecological services by artificial habitats.

## 6. Materials and methods

### 6.1 Study sites

**Fig. 5:**
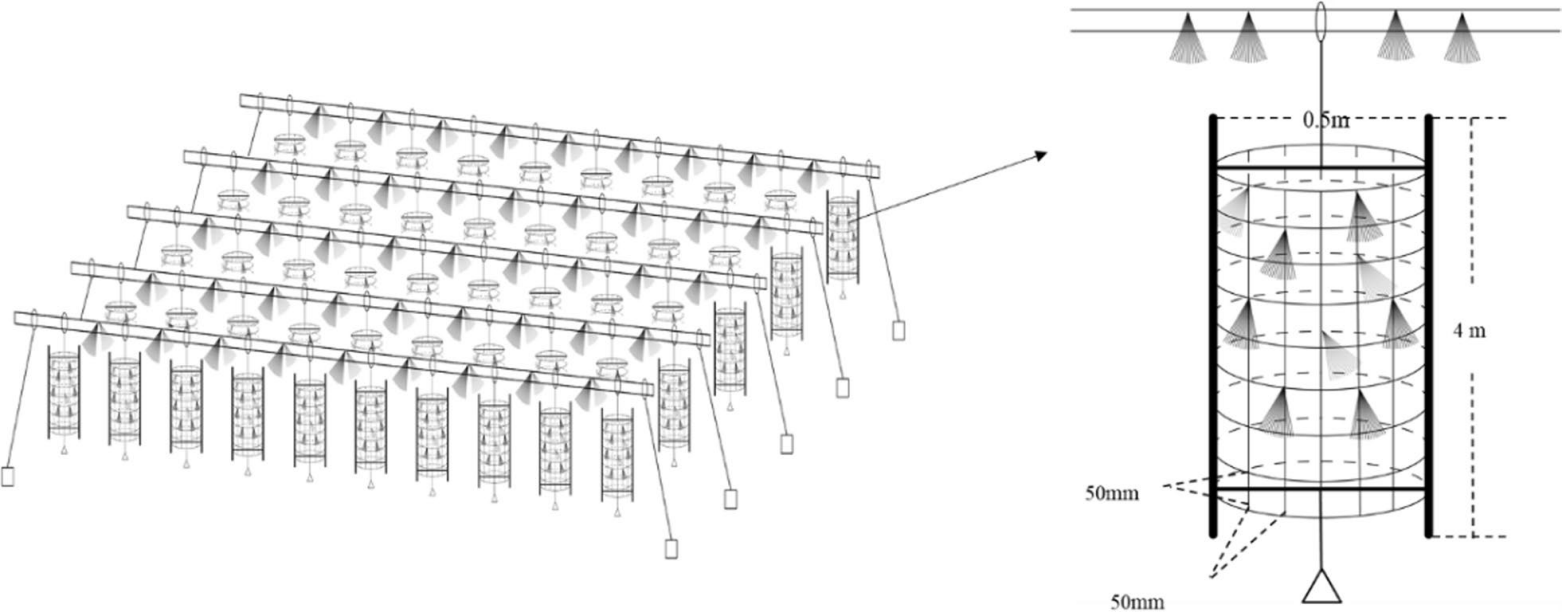
Schematic diagram of the artificial habitats [5]

The artificial habitats were located in the Youjiang River of the Pearl River Basin, China (23.46° N, 106.41° E). An unfed aquaculture program was previously carried out in this river. The artificial habitats were operated in the experimental site of the Youjiang River in December 2015 (Fig. 5) [4, 5].

### 6.2 Sample collection

Samples of the surrounding water, sediment, and tilapia intestines were collected from two distinctive habitats: artificial habitats (AH) and control areas without artificial structures (CW). A total of 180 tilapia were captured, 90 of which came from AH and 90 from CW. The large sample size excluded individual differences in experimental results.

Samples were collected from three random sites for each habitat, and the AH and CW samples were collected at the same time. Thirty samples were taken from each site, totalling 180 intestinal samples (from tilapias captured in artificial habitats AI1, AI2, and AI3, in control areas CI1, CI2, and CI3), 180 water samples (from AH groups AW1, AW2, and AW3, from CW groups CW1, CW2, and CW3) and 180 sediment samples (from AH groups AS1, AS2, and AS3, from CW groups CS1, CS2, and CS3). The entire intestines were sampled to minimize bias caused by the spatial structure [56]. Water samples (1000 mL) were filtered through a 0.22 μm membrane [57]. All the above experimental fish were anesthetized with a high concentration of 50 mg/L tricaine methanesulfonate MS-222 (Beijing Green Hengxing Biological Technology Co). and the surgery and sampling were made after a respiratory arrest.

All animal experimental protocols were approved by the Institutional Animal Care and Use Committee of Sun Yat-sen University, and all methods were carried out following relevant guidelines and regulations.

### 6.3 16 S rDNA sequencing and statistical analysis

V3-V4 hypervariable regions were amplified using the 338 F (ACTCCTACGGGAGGCAGCA) and 806R (GGACTACHVGGGTWTCTAAT) primers [58, 59]. The sequencing work was completed by HiSeq2500 at Biomarker Technologies Co., Ltd. (Beijing, China). The datasets analysed during the current study are available in the NCBI’s GenBank Sequence Read Archive (PRJNA941103).

The diversity index was determined from the OTUs to compare the bacterial community diversity in all groups [60]. The bacterial diversity and OTU richness of habitats and intestines were compared by Kruskal–Wallis tests (*P* < 0.05). PCoA analysis was used to visualize the difference in groups by the R program. BMKCloud (https://international.biocloud.net/zh/dashboard) can be used to analyse Venn diagrams. LEfSe analysis was performed to identify indicator species also using the online BMKCloud.

## Data Availability

All sequence data have been uploaded to NCBI’s GenBank Sequence Read Archive under accession number PRJNA 941,103.

## References

[1] Wu F (2020). Effects of Dietary Carbohydrate to lipid ratio on growth performance, body composition and serum biochemical indices of genetic improvement of Farmed Tilapia in Growth Mid-Stage. Chin J Anim Nutr.

[2] Zhang Z (2020). Recent Research Progresses of Nutrition and feed Science of Freshwater Fish in China. Chin J Anim Nutr.

[3] Shuai F (2020). Fish diversity and distribution pattern of the pearl river system in guangxi. Acta Hydrobiol Sin.

[4] Zhou L (2018). The structuring role of artificial structure on fish assemblages in a dammed river of the Pearl River in China. Aquat Living Resour.

[5] Guo D (2020). Use of artificial structures to enhance fish diversity in the Youjiang River, a dammed river of the Pearl River in China. Ecol Evol.

[6] Del’Duca A, Cesar DE, Abreu PC (2015). Bacterial community of pond’s water, sediment and in the guts of tilapia (Oreochromis niloticus) juveniles characterized by fluorescent in situ hybridization technique. Aquac Res.

[7] Verdegem M et al. The relation between rearing environment on the development of gut microbiota in juvenile tilapia. 2017

[8] Malka H, Ido I (2017). Fish as hosts of Vibrio cholerae. Front Microbiol.

[9] Peterson MS (2006). Foraging in non-native environments: comparison of Nile Tilapia and Three Co-Occurring native centrarchids in invaded Coastal Mississippi Watersheds. Environ Biol Fish.

[10] Pratte ZA (2018). The gills of reef fish support a distinct microbiome influenced by host-specific factors. Appl Environ Microbiol.

[11] Tran NT (2018). Altered gut microbiota associated with intestinal disease in grass carp (*Ctenopharyngodon idellus*). World J Microbiol Biotechnol.

[12] Valdes AM (2018). Role of the gut microbiota in nutrition and health. BMJ.

[13] Pérez T (2010). Host-microbiota interactions within the fish intestinal ecosystem. Mucosal Immunol.

[14] Nayak SK (2010). Role of gastrointestinal microbiota in fish. Aquac Res.

[15] Dehler CE, Secombes CJ, Martin S (2017). Environmental and physiological factors shape the gut microbiota of Atlantic salmon parr (Salmo salar L). Aquaculture.

[16] Vadstein O (2018). Managing the microbial community of marine fish larvae: a holistic perspective for larviculture. Front Microbiol.

[17] Giatsis C (2015). The impact of rearing environment on the development of gut microbiota in tilapia larvae. Rep.

[18] Eichmiller JJ (2016). Environment shapes the fecal microbiome of invasive carp species. Microbiome.

[19] Hou D (2018). Comparative analysis of the bacterial community compositions of the shrimp intestine, surrounding water and sediment. J Appl Microbiol.

[20] Sun Y (2020). Bacterial community compositions of crab intestine, surrounding water, and sediment in two different feeding modes of Eriocheir sinensis. Aquaculture Rep.

[21] Bolding B, Bonar S, Divens M (2004). Use of Artificial structure to Enhance Angler benefits in Lakes, ponds, and Reservoirs: a Literature Review. Rev Fish Sci.

[22] Sosa-Cordero E (1998). Artificial shelters for spiny lobster Panulirus argus (Latreille): an evaluation of occupancy in different benthic habitats. J Exp Mar Biol Ecol.

[23] Sherman RL (2002). Artificial reef design: void space, complexity, and attractants. ICES J Mar Sci.

[24] Jones NE, Tonn WM (2004). Enhancing productive capacity in the canadian Arctic: assessing the effectiveness of Instream Habitat Structures in Habitat Compensation. Trans Am Fish Soc.

[25] Hellyer C, Harasti D, Poore A (2011). Manipulating artificial habitats to benefit seahorses in Sydney Harbour, Australia. Aquat Conserv Mar Freshw Ecosyst.

[26] Wills TC, Bremigan MT, Hayes DB (2004). Variable effects of habitat enhancement structures across species and habitats in michigan reservoirs. Trans Am Fish Soc.

[27] Pickering H, Whitmarsh D, Jensen A (1999). Artificial Reefs as a Tool to Aid Rehabilitation of Coastal Ecosystems: investigating the potential. Mar Pollut Bull.

[28] Sandström A, Karås P (2010). Tests of artificial substrata as nursey habitat for young fish. J Appl Ichthyol.

[29] Hojesj J (2015). Addition of structural complexity – contrasting effect on juvenile brown trout in a natural stream. Ecol Freshw Fish.

[30] Zhu W (2020). Response of protist community dynamics and co-occurrence patterns to the construction of artificial reefs: a case study in Daya Bay, China. Sci Total Environ.

[31] Yang X (2019). Effects of artificial reefs on the meiofaunal community and benthic environment - a case study in Bohai Sea, China. Mar Pollut Bull.

[32] Sun F (2019). Insights into the intestinal microbiota of several aquatic organisms and association with the surrounding environment. Aquaculture.

[33] Zhang C (2019). Bacterial diversity in gut of large yellow croaker *Larimichthys crocea* and black sea bream *Sparus macrocephalus* reared in an inshore net pen. Fish Sci.

[34] Wang AR (2018). Progress in fish gastrointestinal microbiota research. Rev Aquac.

[35] Segata N (2011). Metagenomic biomarker discovery and explanation. Genome Biol.

[36] Kuang T (2020). Comparative analysis of microbial communities associated with the gill, gut, and habitat of two filter-feeding fish. Aquaculture Rep.

[37] Bird AR (2010). Resistant starch, large bowel fermentation and a broader perspective of prebiotics and probiotics. Benef Microbes.

[38] Viaud S (2013). The intestinal microbiota modulates the Anticancer Immune Effects of Cyclophosphamide. Science.

[39] Han L (2016). The gut microbiome and degradation enzyme activity of wild freshwater fishes influenced by their trophic levels. Sci Rep.

[40] Huang Z, et al. Multiple bacteria species were involved in hepatopancreas necrosis syndrome (HPNS) of Litopenaeus vannamei. Acta Sci Nat Univ Sunyatseni 2016. 10.13471/j.cnki.acta.snus.2016.01.001

[41] Cheng Y (2017). A comparative study of microbiota from the intestine of chinese mitten crab (*Eriocheir sinensis*) and their culture environment,between rice-crab coculture and crab monoculture models. J Shanghai Ocean Univ.

[42] Huang F (2018). Microbiota assemblages of water, sediment, and intestine and their associations with environmental factors and shrimp physiological health. Appl Microbiol Biotechnol.

[43] Sugita H (1990). The vitamin B12-producing ability of intestinal bacteria isolated from tilapia and channel catfish. Nippon Suisan Gakkaishi.

[44] Leamaster BR (1997). Cold stress-induced changes in the aerobic heterotrophic gastrointestinal tract bacterial flora of red hybrid tilapia. J Fish Biol.

[45] Schofield PJ (2011). Survival, growth and reproduction of non-indigenous *Nile tilapia*, *Oreochromis niloticus* (Linnaeus 1758). I. physiological capabilities in various temperatures and salinities. Mar Freshw Res.

[46] Russell DJ, Thuesen PA, Thomson FE (2012). A review of the biology, ecology, distribution and control of *Mozambique tilapia*, *Oreochromis mossambicus* (Peters 1852) (Pisces: Cichlidae) with particular emphasis on invasive Australian populations. Rev Fish Biol Fish.

[47] Dibaise JK (2008). Gut microbiota and its possible relationship with obesity. Mayo Clin Proc.

[48] Turnbaugh PJ (2008). Diet-Induced obesity is linked to marked but reversible alterations in the mouse distal gut Microbiome. Cell Host Microbe.

[49] Bennett KW, Eley A (1993). Fusobacteria: New taxonomy and related diseases. J Med Microbiol.

[50] Hoffman H (1952). Bacteriology of the fusobacteria: a review. Oral Surg Oral Med Oral Pathol.

[51] Uchii K (2006). Genetic and physiological characterization of the intestinal bacterial microbiota of Bluegill (*Lepomis macrochirus*) with three different feeding habits. Microb Ecol.

[52] Li T (2015). Comparative analysis of the intestinal bacterial Communities in different species of carp by pyrosequencing. Microb Ecol.

[53] Al-Harbi AH, Uddin N (2005). Bacterial diversity of tilapia (*Oreochromis niloticus*) cultured in brackish water in Saudi Arabia. Aquaculture.

[54] Tongtong (2017). Bacterial signatures of “Red-Operculum” Disease in the gut of Crucian Carp (*Carassius auratus*). Microb Ecol.

[55] Xiong J (2017). Integrating gut microbiota immaturity and disease-discriminatory taxa to diagnose the initiation and severity of shrimp disease. Environ Microbiol.

[56] Smith CC (2015). Dietary input of microbes and host genetic variation shape among-population differences in stickleback gut microbiota. ISME J.

[57] Hou D, Zeng S, Liu J (2017). Characterization of Prokaryotic and eukaryotic Microbial Community in Pacific White shrimp ponds. J Aquaculture Res Dev.

[58] Dongwei H (2017). Environmental factors shape water Microbial Community structure and function in shrimp Cultural Enclosure Ecosystems. Front Microbiol.

[59] Paudel Adhikari N (2019). Bacterial community composition and diversity in Koshi River, the largest river of Nepal. Ecol Ind.

[60] Patrick D (2009). Introducing mothur: open-source, platform-independent, community-supported software for describing and comparing microbial communities. Appl Environ Microbiol.

